# Supplementary material for: The Association of Circulating Amino Acids and Dietary Inflammatory Potential with Muscle Health in Chinese Community-Dwelling Older People
Source: Nutrients. 2022 Jun 15;14(12):2471. doi: 10.3390/nu14122471 (PMC9229609; doi:10.3390/nu14122471)
Supplement: Supplementary file 1 [file nutrients-14-02471-s001.zip › nutrients-1724153-supplementary.pdf]

Supplementary Materials

Supplementary Table S1. The association of baseline serum amino acids with the changes of muscular parameters over 4 years.

AAs	Grip strength (kg)	Gait speed (m/s)	Chair stand test (s)	Appendicular muscle mass (kg/m^2)
	Men			
	β estimate (SE)[a]	β estimate (SE)[a]	β estimate (SE)[a]	β estimate (SE)[a]
DII (per one tertile)	−0.332 (0.143)[b]	−0.013 (0.006)[b]	0.075 (0.104)	0.009 (0.010)
Branched chain amino acids				
Valine (per one tertile)	0.008 (0.148)	−0.005 (0.006)	0.010 (0.107)	−0.008 (0.011)
Leucine (per one tertile)	0.037 (0.150)	−0.001 (0.006)	−0.160 (0.107)	0.008 (0.011)
Isoleucine (per one tertile)	0.142 (0.149)	−0.004 (0.006)	−0.120 (0.108)	0.002 (0.011)
Aromatic amino acids				
Phenylalanine (per one tertile)	0.025 (0.147)	−0.003 (0.006)	−0.135 (0.107)	0.007 (0.011)
Tryptophan (per one tertile)	0.197 (0.144)	0.010 (0.006)	0.043 (0.104)	0.014 (0.010)
Tyrosine (per one tertile)	0.369 (0.142)[b]	−0.004 (0.006)	−0.066 (0.103)	0.009 (0.010)
Sulfur amino acids				
Methionine (per one tertile)	0.221 (0.144)	0.000 (0.006)	−0.193 (0.104)	−0.004 (0.010)
tHcy (per one tertile)	0.004 (0.163)	0.001 (0.007)	0.199 (0.118)	−0.007 (0.012)
Cystathionine (per one tertile)	−0.285 (0.152)	0.000 (0.006)	−0.157 (0.110)	−0.012 (0.011)
tCys (per one tertile)	−0.234 (0.157)	0.003 (0.007)	0.265 (0.114)[b]	−0.003 (0.011)
Taurine (per one tertile)	−0.018 (0.147)	0.011 (0.006)	−0.198 (0.106)	−0.012 (0.011)
tGSH (per one tertile)	0.450 (0.144)[b,c]	0.016 (0.006)[b]	−0.259 (0.105)[b]	0.003 (0.010)
	Women			
	β estimate (SE)[a]	β estimate (SE)[a]	β estimate (SE)[a]	β estimate (SE)[a]
DII (per one tertile)	0.071 (0.099)	−0.003 (0.005)	0.172 (0.173)	−0.007 (0.009)
Branched chain amino acids				
Valine (per one tertile)	−0.002 (0.101)	0.000 (0.005)	−0.077 (0.176)	−0.009 (0.010)

Leucine (per one tertile)	0.044 (0.102)	−0.003 (0.006)	−0.095 (0.179)	0.000 (0.010)
Isoleucine (per one tertile)	−0.062 (0.102)	−0.007 (0.005)	0.219 (0.178)	−0.008 (0.010)
Aromatic amino acids				
Phenylalanine (per one tertile)	0.117 (0.101)	0.005 (0.005)	−0.076 (0.176)	0.014 (0.009)
Tryptophan (per one tertile)	−0.025 (0.100)	0.005 (0.005)	−0.331 (0.173)	−0.022 (0.009)[b]
Tyrosine (per one tertile)	0.033 (0.100)	−0.003 (0.005)	−0.157 (0.173)	−0.003 (0.009)
Sulfur amino acids				
Methionine (per one tertile)	0.151 (0.100)	−0.009 (0.005)	0.038 (0.174)	−0.005 (0.009)
tHcy (per one tertile)	−0.118 (0.115)	−0.007 (0.006)	0.021 (0.200)	0.012 (0.011)
Cystathionine (per one tertile)	−0.138 (0.108)	−0.006 (0.006)	0.050 (0.187)	−0.001 (0.010)
tCys (per one tertile)	−0.282 (0.109)[b]	0.000 (0.006)	0.015 (0.190)	0.016 (0.010)
Taurine (per one tertile)	−0.073 (0.1)	−0.011 (0.005)[b]	0.045 (0.173)	−0.002 (0.009)
tGSH (per one tertile)	0.006 (0.100)	−0.006 (0.005)	0.087 (0.175)	0.025 (0.009)[b]

DII: dietary inflammatory index; OR: odds ratio; CI: confidence interval; SE: standardized error. tHcy: total homocysteine; tCys; total cysteine; tGSH: total glutathione. [a] in sub-fully adjusted model C: adjusted for baseline age, corresponding measurement, estimated glomerular filtration rate, current smoking, physical activity level, previous fracture, hypertension, diabetes, chronic obstructive lung disease, cardiovascular disease, rheumatoid arthritis, nonsteroidal anti-inflammatory agent use and osteoporosis medication. [b] indicates for significant value (P <0.05). [c] indicates for significant value (P <0.004) for amino acids.
